# Comparative evaluation of somatostatin and CXCR4 receptor expression in different types of thyroid carcinoma using well-characterised monoclonal antibodies

**DOI:** 10.1186/s12885-022-09839-z

**Published:** 2022-07-07

**Authors:** Max Czajkowski, Daniel Kaemmerer, Jörg Sänger, Guido Sauter, Ralph M. Wirtz, Stefan Schulz, Amelie Lupp

**Affiliations:** 1 Institute of Pharmacology and Toxicology, Jena University Hospital, Friedrich Schiller University Jena, Drackendorfer Str. 1, D-07747 Jena, Germany; 2 Department of General and Visceral Surgery, Zentralklinik Bad Berka, Bad Berka, Germany; 3 Laboratory of Pathology and Cytology Bad Berka, Bad Berka, Germany; 4 Institute of Pathology, University Medical Center, Hamburg-Eppendorf, Germany; 5 STRATIFYER Molecular Pathology GmbH Köln, Köln, Germany

**Keywords:** Thyroid cancer, Somatostatin receptor, Chemokine receptor, CXCR4, Immunohistochemistry

## Abstract

**Background:**

Papillary and follicular thyroid carcinomas can be treated surgically and with radioiodine therapy, whereas therapeutic options for advanced stage IV medullary and for anaplastic tumours are limited. Recently, somatostatin receptors (SSTs) and the chemokine receptor CXCR4 have been evaluated for the treatment of thyroid carcinomas, however, with contradictory results.

**Methods:**

The expression of the five SSTs and of CXCR4 was assessed in 90 samples from 56 patients with follicular, papillary, medullary, or anaplastic thyroid carcinoma by means of immunohistochemistry using well-characterised monoclonal antibodies. The stainings were evaluated using the Immunoreactivity Score (IRS) and correlated to clinical data. In order to further substantiate the immunohistochemistry results, in serial sections of a subset of the samples receptor expression was additionally examined at the mRNA level using qRT-PCR.

**Results:**

Overall, SST and CXCR4 protein expression was low in all four entities. In single cases, however, very high IRS values for SST2 and CXCR4 were observed. SST2 was the most frequently expressed receptor, found in 38% of cases, followed by SST5 and SST4, found in 14 and 9% of tumours, respectively. SST1 and SST3 could not be detected to any significant extent. CXCR4 was present in 12.5% of medullary and 25% of anaplastic carcinomas. Expression SST3, SST4, SST5 and CXCR4 was positively correlated with expression of the proliferation marker Ki-67. Additionally, a negative interrelationship between SST4 or SST5 expression and patient survival and a positive association between SST3 expression and tumour diameter were observed. qRT-PCR revealed a similar receptor expression pattern to that seen at the protein level. However, probably due to the low overall expression, no correlation was found for the SSTs or the CXCR4 between the IRS and the mRNA values.

**Conclusions:**

SST- or CXCR4-based diagnostics or therapy in thyroid carcinomas should not be considered in general but may be feasible in single cases with high levels of expression of these receptors.

## Background

Thyroid cancer is the most common endocrine neoplasm, accounting for 90% of all endocrine tumours. It ranks ninth in incidence among all cancers worldwide; in 2020, the incidence rates were 10.1 per 100,000 women and 3.1 per 100,000 men [1]. Risk factors include radiation exposure at a young age, excess body weight, hormonal exposures, certain environmental pollutants, and family history [2]. Based on their histopathological characteristics, thyroid malignancies are divided into the four main subtypes papillary thyroid carcinoma (PTC), follicular thyroid carcinoma (FTC), medullary thyroid carcinoma (MTC), and anaplastic thyroid carcinoma (ATC); very rare variants include primary thyroid lymphoma or sarcoma. Of the main subtypes, PTC is predominant and accounts for 80% of all thyroid carcinomas, followed by FTC, accounting for 10–20% of thyroid malignancies [3]. During the past 40 years, the incidence of thyroid cancer, particularly PTC, has risen, which has been attributed to improved imaging modalities and to increasing prevalence of certain risk factors [1, 4].

Whereas PTC and FTC can be successfully treated surgically and with radioiodine therapy, therapeutic options for advanced stage IV MTC and for ATC are limited, and 5-year survival rates are about 28% and 5–7%, respectively, despite improvements in therapy in recent years [5–8]. Therefore, new treatment options are still necessary.

Many tumour entities overexpress receptors for regulatory peptides, which can serve as the molecular basis for targeted diagnostics and treatment modalities. Well-known examples are well-differentiated G1 or G2 gastroenteropancreatic neuroendocrine tumours that overexpress somatostatin receptors (SSTs) [9, 10] and aggressive tumours such as small-cell lung cancer, lymphomas, or G3 gastroenteropancreatic neuroendocrine tumours that overexpress the chemokine receptor CXCR4 [9–12]. Accordingly, in neuroendocrine neoplasms an inverse expression of the SSTs and CXCR4 with increasing malignancy of the tumours has been observed [9, 10]. SST and CXCR4 expression has also been evaluated in thyroid carcinomas, but with contradictory results regarding both the extent of their expression and their correlations with clinical data such as tumour size or stage or patient outcomes (an overview of studies on SST and CXCR4 expression in thyroid carcinomas during the past 20 years is given in Tables 1 and 2). These differences between studies might be due to the wide variety of poly- and monoclonal antibodies and the different rating methods used in the immunohistochemical investigations. In addition, many studies were limited by high rates of background staining and by the observation of only cytoplasmic or even nuclear staining for these membrane-bound receptors.

**Table 1:** Immunohistochemical studies of somatostatin receptor expression in thyroid carcinomas

Study	Samples (n)	Entities tested	SST subtypes	Type of antibody	Positivity (%)	Type of staining	Correlations with clinical data
Papotti et al. 2001 [13]	51	MTC	SST1–5	rabbit polyclonal (self-generated)	SST1: 49%, SST2: 43%, SST3: 47%, SST4: 4%, SST5: 57%	cell membrane and cytoplasm	no correlations with age, sex, tumour size or stage, histological type or outcome
Druckenthaner et al. 2007 [14]	8	7 PTC, 1 FTC	SST2	rabbit polyclonal (Gramsch)	87%	cell membrane	–
Pisarek et al. 2009 [15]	4	2 PTC, 2 PDC	SST1–5	rabbit polyclonal (Gramsch)	SST1: 88.8%, SST2: 44.4%, SST3: 55.5%, SST4: 11.2%, SST5: 33.3%	cell membrane and cytoplasm	–
Müssig et al. 2012 [16]	93	67 PTC, 26 FTC	SST1–5	SST2: rabbit monoclonal (clone UMB1) (Abcam); SST1, SST3, SST4, SST5: rabbit polyclonal (Gramsch)	SST1: 29%, SST2: 19%, SST3: 19%, SST4: 25%, SST5: 15%	cytoplasm	SST2 expression associated with good prognosis
Pazaitou-Panayiotou et al. 2012 [17]	47 (TMA)	38 PTC, 4 FTC, 2 ATC, 3 HTC	SST1–5	rabbit polyclonal (self-generated)	SST1: 75%, SST2: 100%, SST3: 100%, SST4: 38%, SST5: 75%	cell membrane and cytoplasm	no correlations found with tumour size or any other clinicopathological parameters
Woelfl et al. 2014 [18]	87	52 PTC, 24 FTC, 6 MTC, 2 PDC, 3 ATC	SST2, SST5	rabbit monoclonal (clones UMB1, UMB4) (Abcam)	SST2: 11.49%, SST5: 86.20%	cell membrane	SST5 expression associated with good prognosis
Herac et al. 2016 [19]	97	MTC	SST2, SST5	Rabbit monoclonal (clones UMB1; UMB4) (Abcam)	SST2: 66%, SST5: 37.5%	cell membrane and cytoplasm	SST2: positive correlation with presence of lymph node MTS, locally advanced stage, desmoplasia and Ki-67 index; SST5: positive correlation with locally advanced stage and desmoplasia
Kendler et al. 2017 [20]	42	MTC	SST1, SST2, SST3, SST5	rabbit monoclonal (clones UMB7, UMB1, UMB5, UMB4) (Abcam)	SST1: 45.2%, SST2: 28.6%, SST3: 81%, SST5: 54.8%	cell membrane and cytoplasm	positive correlation between SST1 expression and response to initial surgery
de Vries et al. 2018 [21]	114 (TMA)	MTC + 34 MTS	SST2	rabbit monoclonal, clone unknown (Biotrend)	50.9% of PT	cell membrane	positive correlation with overall survival; different expression in PT and MTS
Carmona Matos et al. 2019 [22]	97 (TMA)	57 PTC, 28 FTC, 10 ATC, 2 PDC	SST2	rabbit monoclonal, clone UMB1 (Abcam)	n/s	nuclear and cytoplasm	–
Thakur et al. 2021 [23]	84 (TMA)	39 PTC, 19 FTC, 6 PDC, 4 HTC, 16 MTC	SST2	rabbit polyclonal (ab9550) (Abcam)	SST2: 50% high expression	n/s	–

*ATC* Anaplastic thyroid cancer, *FTC* Follicular thyroid cancer, *HTC* Hürthle  cell thyroid cancer, *MTC* Medullary thyroid cancer, *MTS* Metastasis/metastases; n/s, not specified, *PDC* Poorly differentiated thyroid carcinoma, *PT* Primary tumour, *PTC* Papillary thyroid cancer, *SST* Somatostatin receptor, *TMA* Tissue microarray

**Table 2:** Immunohistochemical studies of CXCR4 expression in thyroid carcinomas

Study	Samples (n)	Entities tested	Type of antibody	Positivity (%)	Type of staining	Correlations with clinical data
Castellone et al. 2004 [24]	19	PTC	mouse monoclonal, clone 12G5 (R&D)	89.5%	n/s	–
De Falco et al. 2007 [25]	33	ATC	mouse monoclonal, clone 12G5 (R&D)	39%	n/s	–
Wagner et al. 2008 [26]	65 (TMA)	PTC	mouse monoclonal, clone 12G5 (R&D)	n/s	cytoplasm	positive correlation with tumour size
Yasuoka et al. 2008 [27]	56	PTC	rabbit polyclonal (Abcam)	60.7%	cytoplasm	higher in patients with lymph node MTS
Gonzalez et al. 2009 [28]	30	PTC	mouse monoclonal, MAB173 (R&D)	90%	cytoplasm	higher in patients with lymph node MTS
He et al. 2010 [29]	50	16 PTC, 18 FTC, 7 MTC, 9 ATC	mouse monoclonal, clone 4G10 (Santa Cruz Biotechnology)	PTC: 68.8%, FTC: 66.7%, MTC: 85.7%, ATC: 100%	mainly nuclear	positive correlation with malignant degree of tumour
Torregrossa et al. 2012 [30]	200	PTC	rabbit polyclonal, ab2047 (Abcam)	80%	cytoplasm	positive correlation with infiltration and BRAF status
Wang et al. 2013 [31]	129	PTC	rabbit polyclonal, ab7199 (Abcam)	97.7%, high expression in 55%	cell membrane and cytoplasm	higher in patients with lymph node MTS
Zhu et al. 2016 [32]	70 (TMA)	40 PTC, 10 FTC, 10 MTC, 10 ATC	mouse monoclonal (Abcam)	PTC: 62.5%, FTC: 30%, MTC: 40%, ATC: 40%	cytoplasm	no difference between PTC with and without lymph node MTS
Werner et al. 2017 [33]	86 (TMA)	MTC	mouse monoclonal (Abcam)	n/s (nearly all stained)	cytoplasm	positive correlation with patient age, tumour size, advanced stage and presence of lymph node or distant MTS; higher in lymph node MTS than in PT
Werner et al. 2018 [34]	44 + 10 MTS (TMA)	FTC	mouse monoclonal (Abcam)	n/s (nearly all stained)	cytoplasm	positive correlation with tumour size, advanced stage; negative correlation with overall and recurrence free survival; higher in distant MTS than in PT
Sirakriengkrai et al. 2020 [35]	74	PTC	mouse monoclonal, MAB172 (R&D)	n/s	nuclear	positive correlation with tumour size
Cao et al. 2021 [36]	115	PTC	mouse monoclonal (Abcam)	46.09% low, 53.91% high expression	cytoplasm	trend towards higher expression with age, positive association with capsule invasion, regional MTS and multifocality
Djafar et al. 2021 [37]	43	PTC	mouse monoclonal, clone 4G10 (Santa Cruz Biotechnology)	90.7%	cell membrane and cytoplasm	higher in patients with lymph node MTS

*ATC* Anaplastic thyroid carcinomas, *FTC* Follicular thyroid carcinomas, *MTC* Medullary thyroid carcinomas, *MTS* Metastasis/metastases, n/s Not specified, *PT* Primary tumour, *PTC* Papillary thyroid carcinomas, *TMA* Tissue microarray

The aim of the present study was to comprehensively re-evaluate SST and CXCR4 expression in a large set of thyroid carcinoma samples of all four entities by immunohistochemistry using well-characterised rabbit monoclonal antibodies. These monoclonal antibodies, which have several advantages over polyclonal ones, were previously generated and extensively characterised by our group [38–43] and have been validated by other researchers (e.g., [44–47]).

## Methods

### Tumour specimens

A total of 90 archived formalin-fixed, paraffin-embedded, whole-block tumour samples from 56 patients were included in the present investigation (specifically, 1 sample each from 32 patients, 2 each from 19 patients, 3 each from 2 patients, 4 each from 2 patients, and 6 from 1 patient; 73 samples were from primary tumours, 15 represented metastases, and 2 samples lacked this information). Of the 56 patients, 19 were diagnosed with PTC, 21 with FTC, 8 with MTC, and 8 with ATC (Table 3). The samples were provided by the Institute of Pathology and Cytology Bad Berka (Bad Berka, Germany) and had been surgically removed between 2007 and 2018 at the Department of Thoracic and Vascular Surgery, Zentralklinik Bad Berka (Bad Berka, Germany).

**Table 3:** Patient and tumour characteristics

		PTC	FTC	MTC	ATC	All tumours
**Total no.**		19	21	8	8	56
**Sex (male/female)**		4/15	8/13	5/3	3/5	20/36
**Age at diagnosis**	**mean**	53.6	60.5	58.1	74.1	59.8
**(years)**	**median**	50.4	60.6	60.5	75.8	60.8
**Living/died/unk.**		17/2/0	16/3/2	2/6/0	1/6/1	36/17/3
**Survival of those**	**mean**	153.3	16.8	81.3	4.7	49.5
**who died (months)**	**median**	153.3*	11.2*	95.2	1.6	7.7
**Tumour diameter**	**mean**	2.2 (*n =* 14)	3.0 (*n =* 15)	4.5 (*n =* 4)	6.4 (*n =* 4)	3.2 (*n =* 37)
**(cm)**	**median**	1.8	2.5	3.3	5.5	2.5
**pT (number)**	**1**	9	7	1	0	17
	**2**	6	6	1	0	13
	**3**	2	6	3	1	12
	**4**	0	0	1	6	7
	**unk.**	2	2	2	1	7
**pN (number)**	**0**	8	5	0	0	13
	**1**	1	3	4	1	9
	**unk.**	10	13	4	7	34
**pM (number)**	**0**	7	4	0	2	13
	**1**	1	5	3	2	11
	**unk.**	11	12	5	4	32

**Fig. 1:**
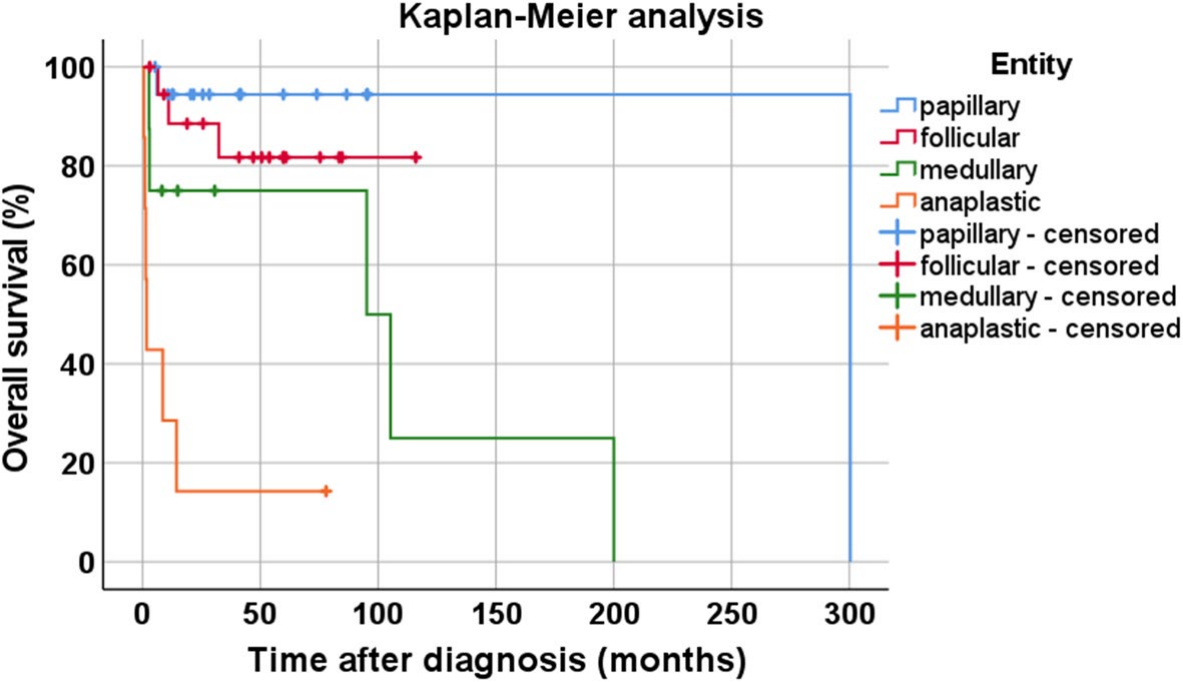
Overall survival of patients with papillary, follicular, medullary, or anaplastic thyroid carcinoma. Breslow test: *p <* 0.001. “Censored”: in the Kaplan-Meier curves the small vertical ticks mark individual patients whose survival times have been “right censored”. These patients did not experience the event of interest (cancer-related death) for the duration of the study and were still alive at the end of the observation period

*ATC* Anaplastic thyroid carcinoma, *FTC* Follicular thyroid carcinoma, *MTC* Medullary thyroid carcinoma, *PTC* Papillary thyroid carcinoma, *pT*, *pN, pM*: TNM classification according to the pathology report; unk.: unknown. *: Please note that in PTC and FTC the median survival time was not reached due to only a few patients who died during the observation period (see Fig. 1)

Additionally, a tissue microarray (TMA) comprising 50 samples from 50 patients with MTC was obtained from the Department of Pathology, University Medical Center, Hamburg-Eppendorf, Germany. For these samples, only data on tumour size (T status) and regional lymph node involvement (N status) were available.

### Patient characteristics

#### Whole-block analysis

In the whole-block analysis, tumours from 20 male (35.7%) and 36 female patients (64.3%) were evaluated (PTC: 4 males, 15 females; FTC: 8 males, 13 females; MTC: 5 males, 3 females; and ATC: 3 males, 5 females) (Table 3). The mean age at diagnosis was 59.8 years overall (median: 60.8 years; range: 26.5–88.0 years). Patients with ATC had a significantly higher age at diagnosis (mean ± S.E.M.: 74.1 ± 3.4 years) compared with those with PTC (mean ± S.E.M.: 53.6 ± 3.4 years; Mann-Whitney test: *p* = 0.001), FTC (mean ± S.E.M.: 60.5 ± 3.5 years; Mann-Whitney test: *p* = 0.047), or MTC (mean ± S.E.M.: 58.1 ± 2.3 years; Mann-Whitney test: *p* = 0.007) (Table 3). The median overall follow-up time was 32.5 months (minimum: 0.7 months; maximum: 300.4 months). At the end of the follow-up period, 36 patients were still living, and 17 patients had died from their tumour. For 3 patients, no survival data were available. Among the patients who died, the median survival time was 7.7 months overall, 1.6 months for the six out of eight patients who died with ATC, 11.2 months for the three out of 21 patients who died with FTC, 95.2 months for the six out of eight patients who died with MTC, and 153.3 months for the two out of 19 patients who died with PTC (Table 3; Kaplan-Meier survival analysis: Breslow test: *p <* 0.001; Fig. 1).

The median tumour diameter at diagnosis of PTC was 1.8 cm, of FTC 2.5 cm, of MTC 3.3 cm, and of ATC 5.5 cm (Kruskal-Wallis test: *p* = 0.034; pairwise Mann-Whitney tests: PTC vs. FTC: *p* = 0.310; PTC vs. MTC: *p* = 0.185; PTC vs. ATC: *p* = 0.004; FTC vs. MTC: 0.442; FTC vs. ATC: *p* = 0.025; MTC vs. ATC: *p* = 0.343) (Table 3).

Among PTCs, nine neoplasms (47.4%) were classified as T1 (tumour limited to the thyroid; tumour size < 2 cm), six (31.6%) as T2 (tumour limited to the thyroid; tumour size 2–4 cm), and two (10.5%) as T3 (tumour limited to the thyroid; tumour size > 4 cm). In two cases (10.5%), the extent of the primary tumour was unknown. Seven FTC tumours (33.3%) were categorised as T1, six (28.6%) as T2, and six (28.6%) as T3. For two FTC neoplasms (9.5%), the T status was not known. Of the MTCs, one tumour (12.5%) was assigned a T1, one (12.5%) a T2, three (37.5%) a T3, and one a T4 (tumour extending beyond the thyroid) status (12.5%). For two patients (25.0%), no information on T status was available. With regard to ATCs, one tumour (12.5%) was classified as T3 and six (75.0%) as T4, and the T status of one tumour (12.5%) was unknown (Table 3).

Eight PTC patients (42.1%) exhibited no lymph node metastases (MTS) at diagnosis, whereas lymph node MTS were present in one case (5.3%). Lymph node status was unknown for 10 PTC patients (52.6%). Among FTC patients, five (23.8%) had no lymph node MTS and three (14.3%) presented with lymph node MTS at diagnosis; in 13 cases (61.9%), the existence of lymph node MTS was not known. For MTC patients, four (50%) had lymph node MTS at diagnosis, and no information on N status was available for the other four patients (50%). One ATC patient (12.5%) presented with lymph node MTS at diagnosis; for all other ATC patients (87.5%), the existence of lymph node MTS was unknown (Table 3).

No distant MTS (M status) were detected in seven PTC patients (36.8%), whereas one patient (5.3%) had distant MTS at diagnosis. The M status was not known in 11 cases (57.9%). Among FTC patients, four (19.1%) exhibited no distant MTS, but distant MTS was reported in the files for five patients (23.8%), and no information on M status was available for 12 patients (57.1%). Of MTC patients, three (37.5%) had distant MTS at diagnosis, and the M status for five patients (62.5%) was unknown. For ATC, two patients (25.0%) had no distant MTS, two patients (25.0%) presented with distant MTS at diagnosis. No data on M status were available for the other four patients (50.0%) (Table 3).

Regarding interrelationships among the different clinical data, patients who had distant MTS at diagnosis were significantly older than those who did not (mean ± S.E.M.: without distant MTS: 49.2 ± 3.9 years; with distant MTS: 68.0 ± 2.7 years; Mann-Whitney test: *p* = 0.003). Additionally, patients who died during the observation period displayed a significantly higher age and had a significantly greater tumour diameter than those who still were alive (mean age ± S.E.M.: alive: 54.9 ± 2.6 years; dead: 66.9 ± 2.5 years; Mann-Whitney test: *p* = 0.007; mean tumour diameter ± S.E.M.: alive: 2.7 ± 0.4 cm; dead: 6.3 ± 1.5 cm; Mann-Whitney test: *p* = 0.012). Correspondingly, a positive correlation between patient age and tumour diameter (r_sp_ = 0.372, *p* = 0.023) and a negative association between patient age and overall survival (r_sp_ = − 0.376, *p* = 0.006) were noted.

#### Tissue Microarray

On the TMA, 16 samples (32.0%) from male and 34 samples (68.0%) from female MTC patients were analysed. The mean age at diagnosis was 56.5 years (median: 57.0 years; range: 20.0–80.0 years). Of the tumours, 32 (64.0%) were classified as T1, 5 (10.0%) as T2, and 12 (24.0%) as T3; for one patient (2.0%), no information on T status was available. Regarding lymph node MTS, 24 patients (48.0%) had none, whereas 18 patients (36.0%) exhibited a positive N status at diagnosis, and N status for eight patients (16.0%) was not known. Data on the M status of the patients were not available.

### Immunohistochemical analyses

From the paraffin blocks, 4-μm sections were prepared and floated onto positively charged slides. Immunostaining was performed using an indirect peroxidase labelling method, as described previously [48]. Rabbit monoclonal antibodies directed against the respective carboxyl-terminal tails of the different receptors were used to detect SSTs and CXCR4 (detailed information regarding the clones, epitopes, and dilutions of the antibodies is given in Table 4). Sections obtained from normal human pancreas (islets: SST1, SST2, SST3, SST5; exocrine pancreas: SST4), lymph nodes (germinal centres: SST2, SST5, CXCR4, Ki-67), and cortex (SST4) were used as positive controls. For negative controls, the primary antibody was either omitted or adsorbed for 2 hours at room temperature with 10 μg/ml of the peptide used for rabbit immunisations [48]. Additional staining was performed with a mouse monoclonal antibody against the proliferation marker Ki-67 (Table 4). Ki-67 is only expressed during the active G1, S, G2 and M phases of the cell cycle but absent from G0 phase of resting cells. Hence, it provides information about the proportion of actively dividing cells in a tissue.

**Table 4:** Antibodies used for immunohistochemical stainings

Antibody	Clone	Type	Epitope	Supplier	Dilution
**SST1**	UMB-7	rabbit monoclonal	ENLESGGVFRNGTCTSRITTL (residues 377–391)	Abcam, Cambridge, UK	1:25
**SST2**	UMB-1	rabbit monoclonal	ETQRTLLNGDLQTSI (residues 335–369)	Abcam, Cambridge, UK	1:10
**SST3**	UMB-5	rabbit monoclonal	QLLPQEASTGEKSSTMRISYL (residues 398–418)	Abcam, Cambridge, UK	1:20
**SST4**	7H49L61	rabbit monoclonal	CQQEALQPEPGRKRIPLTRTTTF (residues 366–388)	Thermo Fisher Scientific, Waltham, MA, USA	1:500
**SST5**	UMB-4	rabbit monoclonal	QEATPPAHRAAANGLMQTSKL (residues 344–364)	Abcam, Cambridge, UK	1:10
**CXCR4**	UMB-2	rabbit monoclonal	KGKRGGHSSVSTESESSSFHSS (residues 338–359)	Abcam, Cambridge, UK	1:2
**Ki-67**	MIB-1	mouse monoclonal		DAKO, Carpintera, CA, USA	1:50

**Fig. 2:**
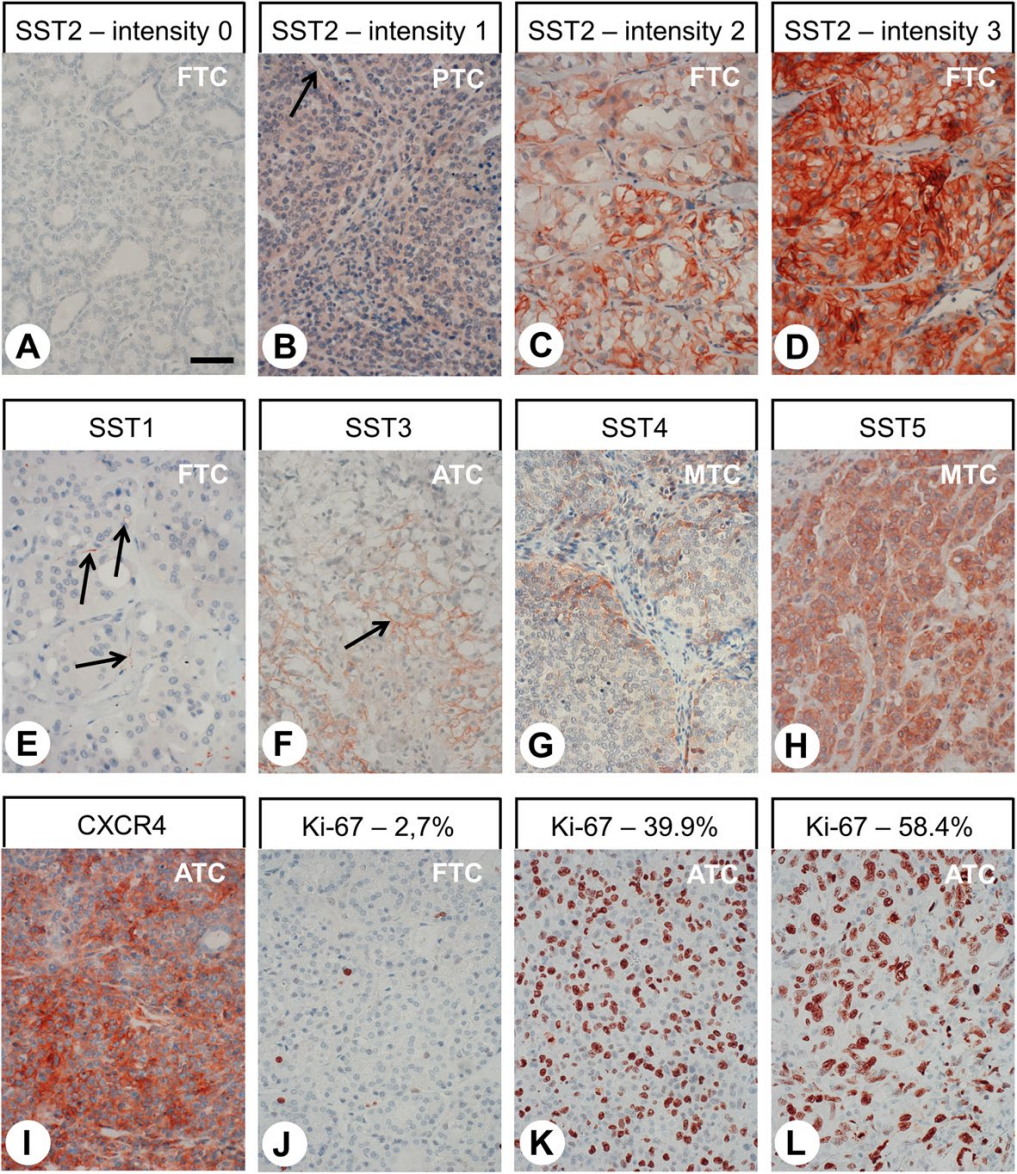
Examples for typical expression patterns of the somatostatin receptors SST1, SST2, SST3, SST4, and SST5 (**A–H**), of the chemokine receptor CXCR4 (I) and of the proliferation marker Ki-67 (**J**–**L**) in thyroid cancer tissues. **A–D**: Examples for a negative staining (intensity 0) and for positive stainings with values of 1, 2, or 3 for the intensity of staining. Immunohistochemistry (red-brown colour), counterstaining with haematoxylin. Scale bar (A–L) = 50 μm. ATC: anaplastic thyroid carcinoma; FTC: follicular thyroid carcinoma; MTC: medullary thyroid carcinoma; PTC: papillary thyroid carcinoma. Arrows: positively stained tumour capillaries

Staining of receptors was scored in all sections using the semi-quantitative Immunoreactivity Score (IRS), as reported by Remmele and Stegner (1987) [49]. The percentage of positively stained tumour cells was stratified into five categories (no positive cells: 0; < 10% positive cells: 1; 10–50% positive cells: 2; 51–80% positive cells: 3; > 80% positive cells: 4) multiplied by one of four values representing the staining intensity of the sample (no staining: 0; weak staining: 1; moderate staining: 2; strong staining: 3; Fig. 2A–D). Thus, IRS values ranging from 0 to 12 were obtained. Tumour samples with an IRS value ≥3 for a given receptor were considered positive for that receptor. For patients with more than one tumour slide, the arithmetic mean was calculated from the IRS values of all slides for that patient, including both primary tumour and MTS (per patient analysis). The antibodies against SSTs and CXCR4 produced distinct immunostaining of not only the plasma membrane of tumour cells but also the cytoplasm, indicative of receptor internalisation due to agonist stimulation (Fig. 2A–I). Both types of staining (cytoplasmic and cell surface) were evaluated equally. With respect to Ki-67 staining, the percentage of positive nuclei was determined. All immunohistochemical stainings were evaluated by two independent, blinded investigators (MC, AL). In the case of discrepant scores, the final decision was reached by consensus.

### Quantitative real-time polymerase chain reaction (qRT-PCR)

In cooperation with STRATIFYER Molecular Pathology, Cologne, Germany, one adjacent paraffin section each from the immunohistochemical slides from 39 patients (12 PTC, 12 FTC, 7 MTC, and 8 ATC) was analysed for SST and CXCR4 mRNA expression. The sections were purified and mRNA was isolated using a standardized isolation method based on magnetic beads [Extraction-XL (96) RNA 2.0 kit; STRATIFYER Molecular Pathology, Cologne, Germany] as previously described [11, 48, 50]. Following extraction, multiplex TaqMan real-time PCR was performed using the SuperScript III Platinum One-Step real-time RT-PCR kit and the Platinum Taq DNA polymerase (Invitrogen, Karlsruhe, Germany). Primers and probes for the SST isoforms and CXCR4 were created by STRATIFYER Molecular Pathology and obtained from Eurogentec (Seraing, Belgium). As a control, the housekeeping gene *CALM2* (calmodulin 2) was used [51]. Furthermore, a no-template control and a human reference RNA (Agilent Technologies, Böblingen, Germany) were measured. Analyses were performed on an Mx3005P apparatus using the MxPro version 4.10d software (Agilent Technologies, Böblingen, Germany). After 40 cycles (50 min at 30 °C, 2 min at 95 °C [15 s at 95 °C, 45 s at 60 °C] × 40), a logarithmic analysis at a threshold of 50 was done. Data were normalised as follows: dCt (Norm) = 40 - ΔCt (Ct (receptor) - Ct (CALM2)). Finally, dCt values ≥19.00 were obtained, which were used for the subsequent calculations.

### Statistical analysis

SPSS 25.0.0.0 (IBM, Armonk, NY, USA) was used for statistical analysis. Because the data were not normally distributed (Kolmogorov-Smirnov-test), Kruskal-Wallis test, Mann-Whitney test, Chi-square test, Kendall’s τ-b test or Spearman’s rank correlation was performed. For survival analysis, the Kaplan-Meier method with a log-rank test was used. *P* values ≤0.05 were considered statistically significant.

## Results

### Whole-block samples

#### Immunohistochemistry

Representative examples of the immunostainings for SSTs, CXCR4 and Ki-67 are depicted in Fig. 2. Overall, the antibodies against SSTs and CXCR4 produced distinct immunostaining of the plasma membrane but also of the cytoplasm of the tumour cells. Apart from (and independent of) staining in the tumour cells, strong expression of the receptors was found on the tumour capillaries in many cases (Fig. 2B, E, F).

**Fig. 3:**
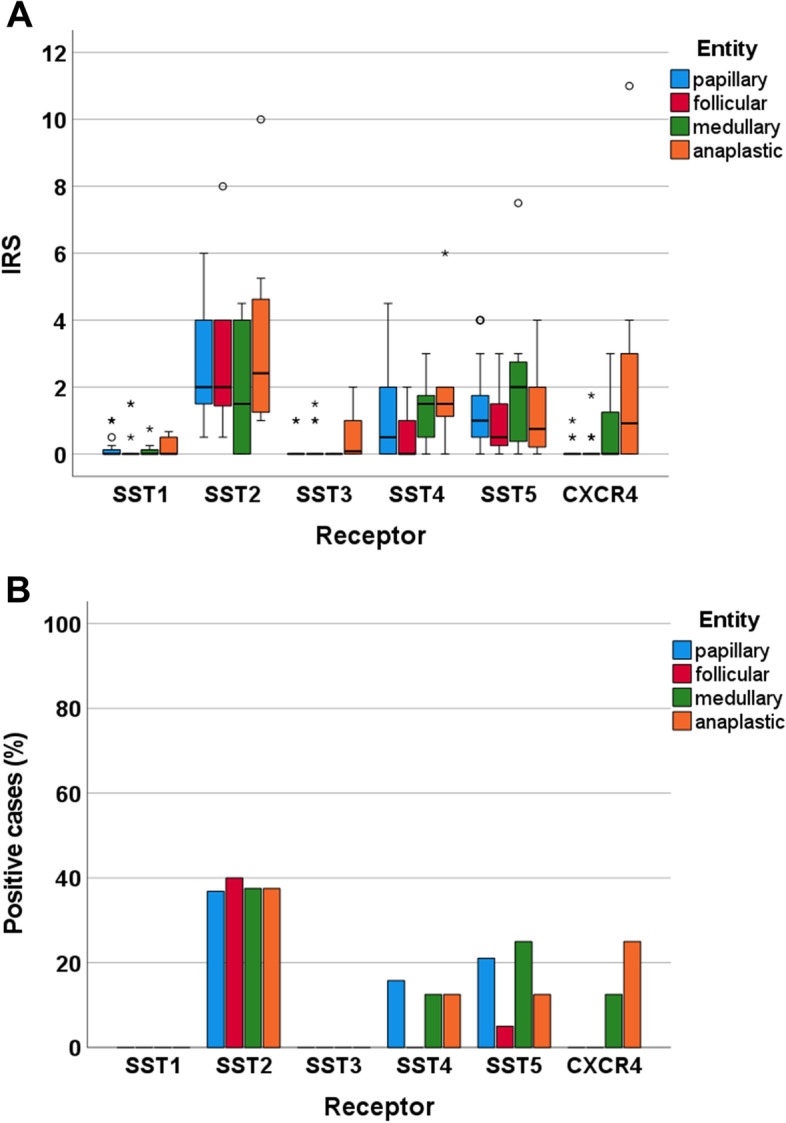
Expression profiles of the somatostatin receptor (SST) subtypes SST1, SST2, SST3, SST4, and SST5 and the chemokine receptor CXCR4 in whole-block thyroid cancer samples at the protein level, separated by the four tumour entities. (A): Box plots of the expression levels (Immunoreactivity Score [IRS] values) as determined by immunohistochemistry of the SSTs and CXCR4. Median values, upper and lower quartiles, minimum and maximum values, and outliers are depicted. The outliers are defined as follows: circles: mild outliers, 1.5–3 times more extreme than the upper or lower quartiles; asterisks: extreme outliers, > 3 times as extreme as the upper or lower quartiles. (B): Percentage of tumours positive for the different SSTs and CXCR4. Only tumours with an IRS ≥3 were considered positive

Figure 3 shows the IRS values and the numbers of the thyroid cancer samples, subdivided according to the four entities, that were positive (IRS ≥3) for the different SSTs and CXCR4. For all receptors, but especially SST2, SST4, SST5, and CXCR4, expression levels varied considerably among the individual patients, which is reflected by the large number of outliers and the length of the boxes and whiskers depicted in Fig. 3A. However, SST and CXCR4 expression levels were generally very low in the thyroid cancer samples investigated. Overall, SST2 was the most prominently expressed receptor, followed by SST5, SST4, and CXCR4. SST2 was present in 38% of the samples overall (IRS ≥3), but the median IRS for SST2 across all four entities was only 2.0. Between the different entities, the median IRS varied between 1.5 (MTC) and 2.4 (ATC). SST5 was found in 5–20% of tumours, with a median IRS of 1.0 across all four entities; IRS for SST5 varied between 0.5 (FTC) and 2 (MTC). SST4 was expressed in 0–16% of the carcinoma samples. Here, the median IRS was 1.0 across all four entities and varied between 0 (FTC) and 1.5 (MTC, ATC). For SST1 and SST3, none of the tumour samples reached an IRS of 3, the cut-off value for receptor positivity. Accordingly, the median IRS for these receptors in each entity and across all entities was 0. CXCR4 was present in 12.5% of MTC and 25% of ATC samples. The median IRS for CXCR4 in ATC was 1.0 but for all other entities it was 0. Thus, across all tumours, the median IRS for CXCR4 was also 0. Significant differences between the four thyroid carcinoma entities with regard to receptor expression were only observed for SST4 and CXCR4. ATC displayed significantly higher SST4 IRS values than did FTC (Mann-Whitney test: *p* = 0.013) and significantly higher CXCR4 IRS values than did PTC (Mann-Whitney test: *p* = 0.034) or FTC (Mann-Whitney test: *p* = 0.041).

**Fig. 4:**
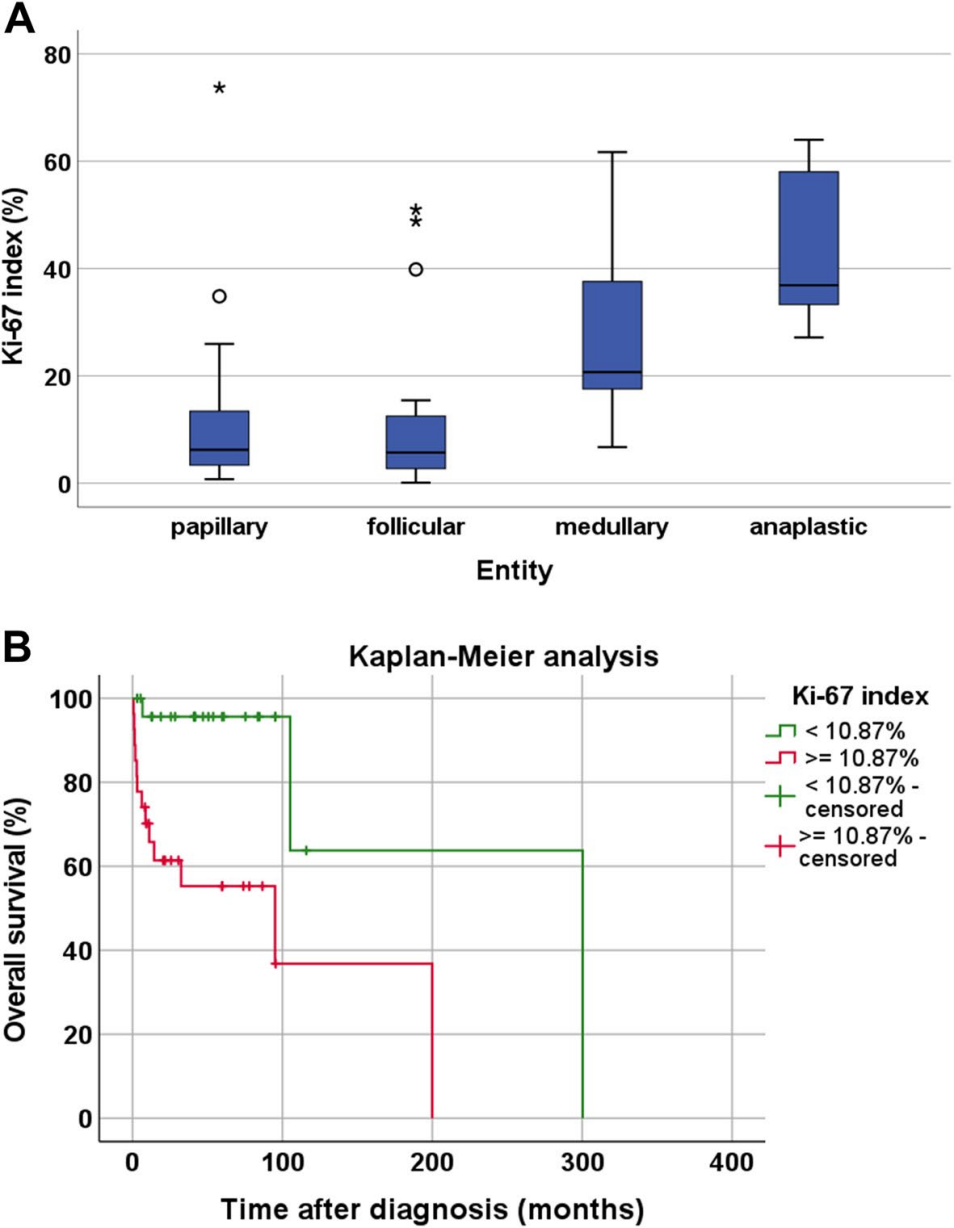
Ki-67 expression at the protein level in whole-block thyroid cancer samples and influence of the level of Ki-67 expression on patient overall survival. **A**: Box plots of the of the Ki-67 index (%) as determined by immunohistochemistry, separated by the four tumour entities. Median values, upper and lower quartiles, minimum and maximum values, and outliers are depicted. The outliers are defined as follows: circles: mild outliers, 1.5–3 times more extreme than the upper or lower quartiles; asterisks: extreme outliers, > 3 times as extreme as the upper or lower quartiles. **B**: Overall survival of patients with thyroid carcinoma in dependence of the Ki-67 level of the tumour. The overall median Ki-67 value of 10.87% was set as the cut-off value between low and high Ki-67 expression. Log-rank test: *p* = 0.001; Breslow test: *p* = 0.002. “Censored”: in the Kaplan-Meier curves the small vertical ticks mark individual patients whose survival times have been “right censored”. These patients did not experience the event of interest (cancer-related death) for the duration of the study and were still alive at the end of the observation period

The median Ki-67 index (i.e., the percentage of Ki-67 positive nuclei out of all nuclei) for all samples examined was 10.87. Differences in expression of Ki-67 were observed between PTC and FTC on the one hand (median Ki-67 index of 6.2 and 5.7, respectively) and MTC and ATC on the other (median Ki-67 index of 20.7 and 36.9, respectively) (Fig. 4A). These differences were statistically significant (Kruskal-Wallis test: *p <* 0.001; pairwise Mann-Whitney tests: PTC vs. MTC: p = 0.013; PTC vs. ATC: *p <* 0.001; FTC vs. MTC: *p* = 0.008; FTC vs. ATC: *p <* 0.001).

SST2 immunostaining was positively correlated with SST1 (r_sp_ = 0.361, *p* = 0.006) and SST5 expression (r_sp_ = 0.330, p = 0.013); and SST3 immunostaining with SST4 expression (r_sp_ = 0.346, *p* = 0.009) (Table 5). Furthermore, SST3 (r_sp_ = 0.351, p = 0.008), SST4 (r_sp_ = 0.398, *p* = 0.002), SST5 (r_sp_ = 0.277, *p <* 0.038), and CXCR4 expression (r_sp_ = 0.298, *p* = 0.028) displayed a positive interrelationship with Ki-67 index (Table 5).

**Table 5:** Correlations between expression intensities of the different SSTs, CXCR4 and Ki-67 in the thyroid carcinoma cases investigated

		SST2	SST3	SST4	SST5	CXCR4	Ki-67
**SST1**	r (*p*)	**0.361 (0.006)**	0.015 (0.914)	0.210 (0.120)	0.133 (0.328)	−0.174 (0.199)	0.211 (0.118)
**SST2**	r (*p*)		0.091 (0.505)	0.258 (0.055)	**0.330 (0.013)**	−0.015 (0.913)	0.216 (0.110)
**SST3**	r (*p*)			**0.346 (0.009)**	−0.039 (0.778)	0.119 (0.382)	**0.351 (0.008)**
**SST4**	r (*p*)				0.216 (0.109)	0.204 (0.131)	**0.398 (0.002)**
**SST5**	r (*p*)					0.103 (0.448)	**0.277 (0.038)**
**CXCR4**	r (*p*)						**0.298 (0.028)**

r: correlation coefficient (Spearman); p: *p* value; *n =* 56; significant correlations (*p <* 0.05) are marked in bold

Positive associations were observed between SST3 expression and patient age (r_sp_ = 0.264, *p* = 0.003), tumour diameter (r_sp_ = 0.358, *p* = 0.030) or T stage (τ = 0.371, *p* = 0.004), and between SST4 expression and patient age (r_sp_ = 0.268, *p* = 0.030). A negative correlation was observed between SST5 expression and overall survival (r_sp_ = − 0.293, *p* = 0.032). Patients who were still alive at the end of the observation period showed significantly lower SST4 IRS values than did those who had died (mean ± S.E.M.: alive: 0.79 ± 0.18; deceased: 1.67 ± 0.37; Mann-Whitney test: *p* = 0.017). In the respective Kaplan-Meier analyses, however, only a tendency towards a worse outcome in patients with SST4-positive tumours (IRS ≥ 3) was noted (Log-rank test: *p* = 0.135; Breslow test: *p* = 0.129). Similar results were obtained when using the median IRS value of 1.0 as cut-off (Log-rank test: *p* = 0.127; Breslow test: *p* = 0.118) or an IRS value of 0.415 determined by ROC analysis to represent the optimal threshold for discrimination between groups (Log-rank test: *p* = 0.103; Breslow test: *p* = 0.088). Furthermore, a positive interrelationship between CXCR4 expression and patient age was observed (r_sp_ = 0.176, *p* = 0.050). Between Ki-67 expression and patient age (r_sp_ = 0.343, *p* = 0.010), tumour diameter (r_sp_ = 0.626, *p <* 0.001) or T stage (τ = 0.432; *p <* 0.001) a positive correlation was noted, but a negative association between Ki-67 index and patient overall survival (r_sp_ = − 0.270, p = 0.050). The latter interrelationship was verified by a Kaplan-Meier analysis with the cut-off between high and low Ki-67 expression set at the overall median Ki-67 value of 10.87% (Fig. 4B; Log-rank test: *p* = 0.001; Breslow test: *p* = 0.002). Additionally, patients who presented with lymph node MTS at diagnosis had significantly higher Ki-67 values than did patients without lymph node MTS (mean ± S.E.M.: without lymph node MTS: 7.5% ± 1.8%; with lymph node MTS: 19.7% ± 5.2%; Mann-Whitney test: *p* = 0.030). No other associations between SST, CXCR4, or Ki-67 expression and clinical data were noted.

#### qRT-PCR analysis

**Fig. 5:**
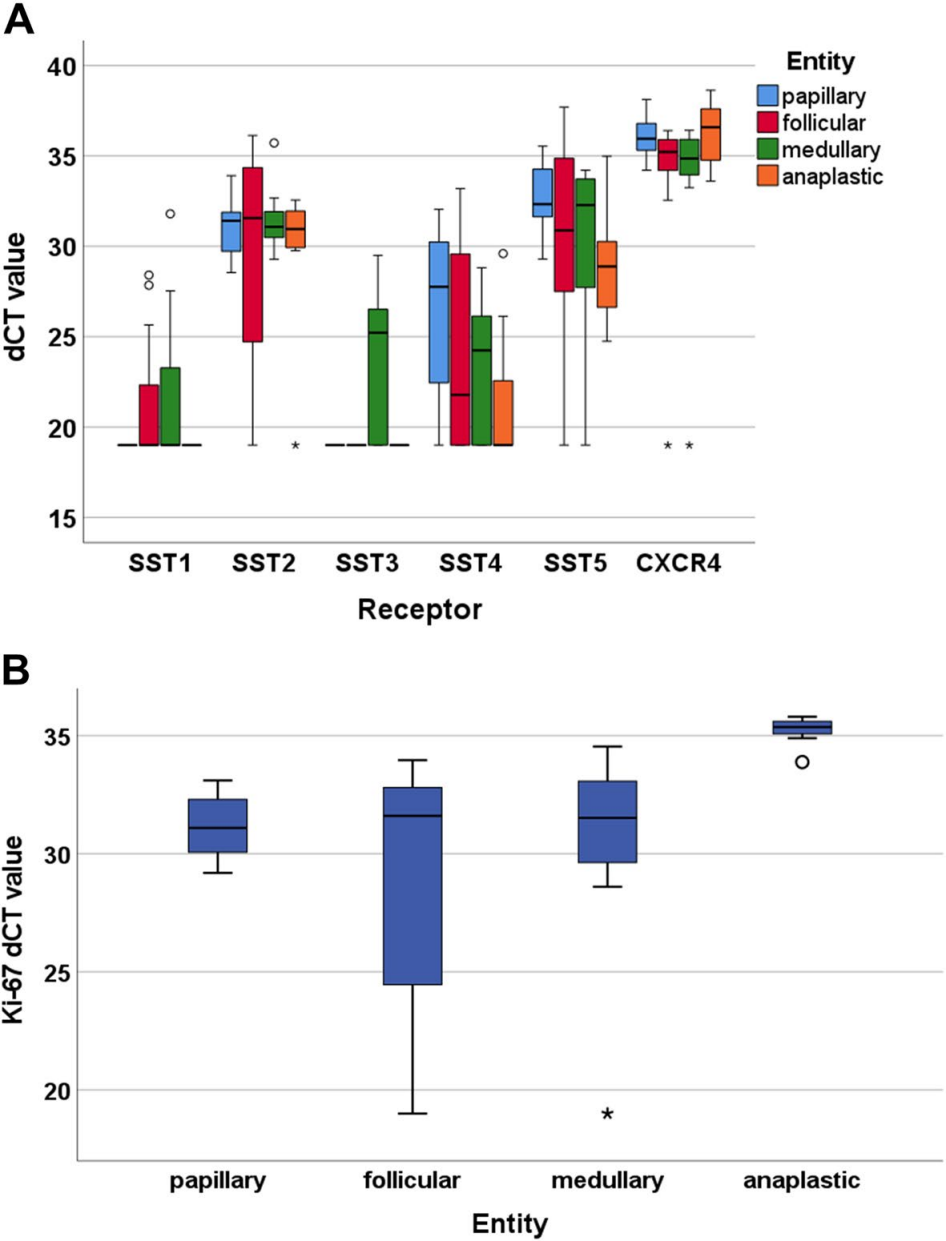
Expression profiles of the somatostatin receptor (SST) subtypes SST1, SST2, SST3, SST4, and SST5 and the chemokine receptor CXCR4 and of the proliferation marker Ki-67 in whole-block thyroid cancer samples at the mRNA level, separated by the four tumour entities. **A**: Box plots of the expression levels (dCT values) as determined by quantitative real-time polymerase chain reaction (qRT-PCR) of the SSTs and CXCR4. **B**: Box plots of the expression levels (dCT values) as determined by qRT-PCR of Ki-67. Median values, upper and lower quartiles, minimum and maximum values, and outliers are depicted. The outliers are defined as follows: circles: mild outliers, 1.5–3 times more extreme than the upper or lower quartiles; asterisks: extreme outliers, > 3 times as extreme as the upper or lower quartiles

At the mRNA level, CXCR4 was the most prominently expressed receptor (overall median dCT value: 35.8), followed by SST5 (overall median dCT value: 31.7), SST2 (overall median dCT value: 31.3), and SST4 (overall median dCT value: 24.6). For SST1 and SST3, the overall median dCT values were only 19, which represents the null value, corresponding to no expression (Fig. 5A). With a median dCT value of 23.5, MTC displayed a higher SST3 mRNA expression than the other three tumour entities with median dCT values of only 19 (pairwise Mann-Whitney tests: PTC vs. MTC: *p* = 0.045; FTC vs. MTC: *p* = 0.045; ATC vs. MTC: *p* = 0.072). With regard to SST4 mRNA expression, a significant difference was found between PTC (median dCT value: 27.8) and ATC (median dCT value: 19) (Mann-Whitney test: *p* = 0.039), and also for SST5, a significant difference in the dCT values was observed between PTC (median dCT value: 32.3) and ATC (median dCT value: 28.9) (Mann-Whitney test: *p* = 0.004). For SST1, SST2, and CXCR4, no difference in the mRNA expression levels between the four entities was noted.

The overall median dCT value for the proliferation marker Ki-67 was 32.1 (Fig. 5B). Whereas Ki-67 mRNA expression levels were similar for PTC (median dCT value: 31.9), FTC (median dCT value: 31.6), and MTC (median dCT value: 31.5), significantly higher values were observed for ATC (median dCT value: 35.4) (pairwise Mann-Whitney tests vs. ATC: *p* ≤ 0.001).

Significant correlations were observed between mRNA expression of SST1 and SST3 (r_sp_ = 0.390, *p* = 0.014), SST4 and SST5 (rs_p_ = 0.674, *p <* 0.001), and CXCR4 and Ki-67 (r_sp_ = 0.398, *p* = 0.012).

No significant associations with the clinical data were noted for the dCT values of SSTs and CXCR4. Similar to the results obtained at the protein level, however, a significant positive interrelationship was found for the dCT values of Ki-67 with patient age (r_sp_ = 0.337, *p* = 0.036), tumour diameter (r_sp_ = 0.506, *p* = 0.008), and T stage (τ = 0.649, *p <* 0.001), and a negative correlation was found for Ki-67 dCT values with patient overall survival (r_sp_ = − 0.374, *p* = 0.023). Ki-67 mRNA values were additionally higher for patients who presented with lymph node MTS at diagnosis than for those who did not (mean ± S.E.M.: without lymph node MTS: 28.7 ± 1.7; with lymph node MTS: 31.3 ± 2.1; Mann-Whitney test: *p* = 0.038). Furthermore, significantly higher Ki-67 dCT values were observed for patients who died during the observation period than for those who were still alive (mean ± S.E.M.: alive: 30.5 ± 0.8; dead: 32.7 ± 1.1; Mann-Whitney test: *p* = 0.012). This result was verified by a Kaplan-Meier analysis when taking the overall median Ki-67 dCT value of 32.1 as the cut-off value between low and high Ki-67 expression (Log-rank test: *p <* 0.001).

Due to their very low overall values, no correlation was observed between mRNA and protein levels for SSTs or CXCR4, but a significant interrelationship was found for Ki-67 (r_sp_ = 0.483; *p* = 0.002).

### Tissue Microarray analysis

**Fig. 6:**
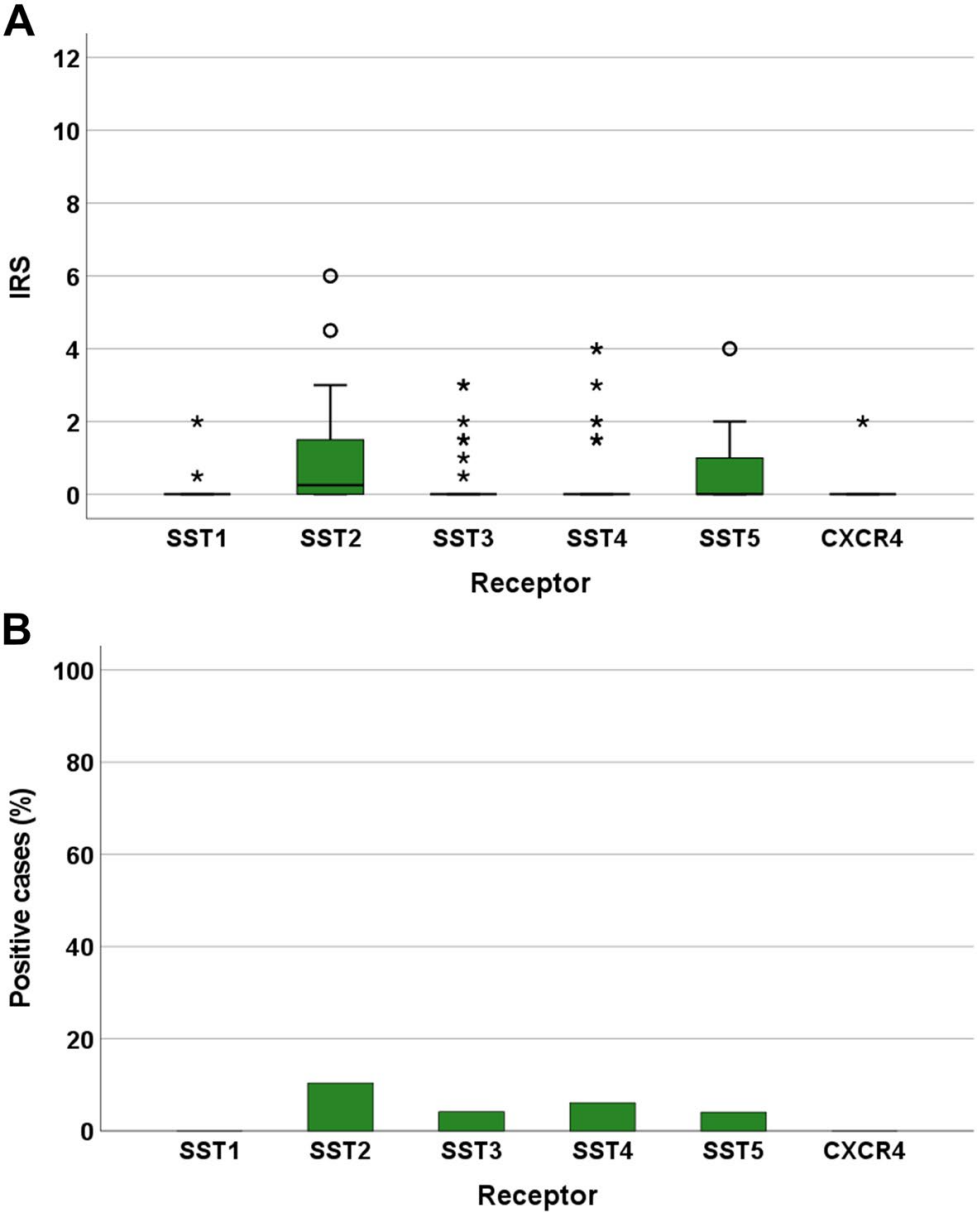
Expression profiles of the somatostatin receptor (SST) subtypes SST1, SST2, SST3, SST4, and SST5 and the chemokine receptor CXCR4 in the medullary thyroid carcinoma (MTC) samples on the tissue microarray (TMA) at the protein level. **A**: Box plots of the expression levels (Immunoreactivity Score [IRS] values) as determined by immunohistochemistry of the SSTs and CXCR4. Median values, upper and lower quartiles, minimum and maximum values, and outliers are depicted. The outliers are defined as follows: circles: mild outliers, 1.5–3 times more extreme than the upper or lower quartiles; asterisks: extreme outliers, > 3 times as extreme as the upper or lower quartiles. **B**: Percentage of MTC on the TMA positive for the different SSTs and CXCR4. Only tumours with an IRS ≥3 were considered positive

The IRS values and the numbers of the MTC samples on the TMA that were positive (IRS ≥3) for SSTs and CXCR4 are depicted in Fig. 6.

As in the whole-block MTC samples, the extent of SST and CXCR4 expression in the TMA tumour samples was very low overall. The median IRS for all receptors was 0. Consequently, for SST5, the IRS values determined for the TMA samples were significantly lower than those determined for the whole-block MTC samples (Mann-Whitney test: *p* = 0.011). The median IRS value for SST2 in the TMA samples was 0.25, which was also lower than that in the whole-block samples. However, this difference was not statistically significant (Mann-Whitney test: *p* = 0.304). Nonetheless, as in the MTC whole-block samples, SST2, SST4 and SST5 showed the highest overall expression also in TMA samples (Fig. 6A).

None of the TMA samples examined had an IRS ≥3 and thus positivity for SST1 or CXCR4. Among the receptors tested, SST2 was found in the most TMA samples (10.4%), followed by SST4 (6.1%), SST3 (4.2%) and SST5 (4.1%) (Fig. 6B).

The median Ki-67 index of the TMA samples was 2.8% (mean: 7.0%), the lowest value found was 0.2%, and the highest was 68.1%. Thus, the Ki-67 index in the TMA samples was overall significantly lower than that in the MTC whole-block samples (Mann-Whitney test: *p <* 0.001).

With the TMA samples, as with the whole-block samples, a significant positive correlation was determined between SST2 and SST4 (r_sp_ = 0.300, *p* = 0.038), between SST2 and SST5 (r_sp_ = 0.500, *p <* 0.001), between SST4 and SST5 (r_sp_ = 0.343, *p* = 0.016), and between SST3 and Ki-67 expression (r_sp_ = 0.330, *p* = 0.024). However, in the TMA samples, no other correlations between the receptors and Ki-67 were found.

As in the whole-block samples, a significant positive correlation between SST3 expression and patient age was noted in the TMA samples (r_sp_ = 0.295, *p* = 0.042), but there were no correlations between Ki-67 expression and clinical data.

## Discussion

In contrast to the majority of the existing data for SSTs and CXCR4 (Tables 1 and 2), our investigations revealed low levels of SST and CXCR4 expression in thyroid carcinoma samples overall. One reason for this discrepancy may be that in the present study well-characterised monoclonal antibodies were used to measure expression levels, whereas in previous investigations a variety of poly- and monoclonal antibodies from various commercial and non-commercial sources was used, which might also explain the huge variability in the SST and CXCR4 expression levels among these studies. We used the IRS to evaluate the expression levels of the receptors in the cancer tissues, taking both the frequency and the intensity of expression into account. Only samples displaying an IRS ≥3 were considered positive for receptor expression. Some previous studies did not describe whether the staining frequency and intensity were both measured or which rating method was used. Because a given receptor must display at least moderately strong expression intensity (i.e., IRS ≥6) to be clinically useful as a target structure, our results indicate that very few patients with thyroid cancer would likely benefit from SST- or CXCR4-based diagnostics or therapy. In this regard, our results correspond well to previous reports that SST-based positron emission tomography (PET) or PET/computed tomography is suitable in only a selected group of thyroid cancer patients [52–57], that somatostatin analogues like octreotide show no beneficial effect in the treatment of thyroid carcinomas [52, 58], and that a very limited number of patients benefits from SST-based peptide receptor radionuclide therapy [23, 52–55]. CXCR4-based diagnostics and therapies have not yet been undertaken in thyroid carcinomas, which also indirectly supports our (largely negative) results.

Whereas no major differences in SST expression were noted between the four thyroid carcinoma entities, CXCR4 expression was significantly higher in highly malignant APC than in well-differentiated PTC and FTC. Additionally, a positive correlation between CXCR4 and Ki-67 expression data was noted. This correlation was expected because, according to the literature, CXCR4 is mainly expressed in highly malignant tumours and is associated with rapid tumour growth, early metastasis, and poor patient outcomes [9–12, 59, 60].

Independent of the presence or absence of expression in the tumour cells, SST and CXCR4 were often strongly expressed on the tumour capillaries. Similar observations have been described for many other tumour entities (e.g., [61–67]). Neo-angiogenesis plays an important role in the development, progression, and metastasis of many types of tumour, including thyroid carcinomas [68, 69]. Therefore, targeting of tumour microvessels using anti-SST or anti-CXCR4 therapies might represent a promising (additional) therapeutic strategy for thyroid carcinomas.

When comparing results from the TMA and the whole-block tumour samples for MTC, generally higher SST and CXCR4 expression rates were observed with the whole blocks. That discrepancy might be due to the high heterogeneity of SST and CXCR4 expression among individual tumours, which was visible in the whole-block tumour samples. Pronounced intra-individual variability in SST and CXCR4 expression is well documented in the literature for many tumour entities (e.g., [10, 11, 64, 66, 67, 70]) and has also been noted for thyroid cancer [17, 19–21]. This variability might have led to an underestimation of SST and CXCR4 expression in the TMA samples in our investigation. Similar observations for these receptors have been made in prostate cancer, although in that study three tissue cylinders were taken per tumour block to compensate for intraindividual heterogeneity of receptor expression [67].

The median Ki-67 index for all samples examined was 10.87, with a gradual increase from well-differentiated PTC and FTC (with a median Ki-67 index around 6) to MTC and eventually ATC (with a median Ki-67 index of about 37). These values correspond well to the data reported in the literature for the different thyroid carcinoma entities [16, 18, 71]. Furthermore, in the present study, a positive association between Ki-67 values and tumour stage and a negative correlation between Ki-67 index and patient outcomes was noted. Thus, as already proposed in the literature, Ki-67 may serve as a valuable prognostic marker in thyroid carcinomas [16, 71, 72].

The IRS values of the SST3, SST4 and SST5 positively correlated with Ki-67 values. For SST3 expression, an additional association with tumour diameter and, for SST4 and SST5 expression, a negative correlation with patient outcomes were observed. Therefore, SST expression may also serve as negative prognostic marker. This finding fits with some of the literature data on SST2 expression [19], but contrasts with others, in which either SST2 or SST5 were presented as positive predictive markers [16, 18, 21].

mRNA values in the present study showed a similar pattern to that seen at the protein level. SST2 and SST5 had the highest mRNA expression among SST subtypes, whereas very low to no mRNA expression was noted for SST1 and SST3. These findings are in concordance with the literature [14, 73, 74]. However, probably due to the low overall expression, no correlation was found for SSTs or CXCR4 between the protein and mRNA levels. By contrast, a clear-cut correlation between protein and mRNA levels was noted for Ki-67. Similar to protein levels, Ki-67 mRNA expression was highest in ATC, and there was a positive correlation between Ki-67 mRNA levels and tumour stage and a negative association between Ki-67 mRNA levels and patient outcomes. Therefore, as has been demonstrated already in the literature [72], both protein and mRNA levels of Ki-67 allow for a prognostic statement in thyroid carcinomas.

## Conclusions

SST and CXCR4 expression levels are generally low in thyroid carcinomas. Therefore, SST- or CXCR4-based diagnostics or therapy in thyroid carcinomas should not be considered in general, although they might be feasible in single cases with high expression of these receptors. Ki-67 expression at both the protein and the mRNA levels represents a valuable prognostic marker in thyroid carcinomas.

## Data Availability

All data generated during this study are included in this published article. The amino acid sequences of the receptors against which the antibodies were raised are publicly available through the Uniprot database: SST1: https://www.uniprot.org/uniprotkb/P30872/entry; SST2: https://www.uniprot.org/uniprotkb/P30874/entry; SST3: https://www.uniprot.org/uniprotkb/P32745/entry; SST4: https://www.uniprot.org/uniprotkb/P31391/entry; SST5: https://www.uniprot.org/uniprotkb/P35346/entry; CXCR4: https://www.uniprot.org/uniprotkb/P61073/entry.

## Abbreviations

ATC: Anaplastic thyroid carcinoma
CXCR4: CXC motif chemokine receptor 4
FTC: Follicular thyroid carcinoma
IRS: Immunoreactivity Score
MTC: Medullary thyroid carcinoma
MTS: Metastases
PTC: Papillary thyroid carcinoma
SST: Somatostatin receptor
sp.: Spearman
TMA: Tissue microarray
